# Eicosapentaenoic Acid Ameliorates Cardiac Fibrosis and Tissue Inflammation in Spontaneously Hypertensive Rats

**DOI:** 10.1016/j.jlr.2022.100292

**Published:** 2022-10-05

**Authors:** Nazli Gharraee, Zhan Wang, Adam Pflum, Danielle Medina-Hernandez, David Herrington, Xuewei Zhu, Giselle C. Meléndez

**Affiliations:** 1 Department of Internal Medicine, Section on Cardiology, Wake Forest School of Medicine, Winston-Salem, North Carolina, USA; 2 Department of Internal Medicine, Section on Molecular Medicine, Wake Forest School of Medicine, Winston-Salem, North Carolina, USA; 3 Department of Pathology, Section on Comparative Medicine, Wake Forest School of Medicine, Winston-Salem, North Carolina, USA

**Keywords:** dietary fat, cytokines, macrophages, omega-3 fatty acids, cardiac remodeling, cardiac hypertrophy, diastolic dysfunction, blood pressure, hypertensive cardiomyopathy, interstitial fibrosis, DBP, diastolic blood pressures, DPA, docosapentaenoic acid, HF, heart failure, HTN, hypertension, IL, Interleukin, LV, left ventricle, LVID, left ventricular internal dimension, SBP, systolic blood pressures, SHR, spontaneously hypertensive rats, TGF, transforming growth factor

## Abstract

Hypertension affects 1 in 3 adults in the United States and leads to left ventricular (LV) concentric hypertrophy, interstitial fibrosis, and increased stiffness. The treatment of cardiac fibrosis remains challenging and empiric. Eicosapentaenoic acid (EPA) is an omega-3 polyunsaturated fatty acid that is highly effective in reducing cardiovascular events in patients and cardiac fibrosis and hypertrophy in animals when administered before pressure overload by promoting the increase of anti-inflammatory M1 macrophages. In this study, we investigated whether EPA mitigates the exacerbation of cardiac remodeling and fibrosis induced by established hypertension, a situation that closely recapitulates a clinical scenario. Twelve-week-old spontaneously hypertensive rats were randomized to eat an EPA-enriched or control diet for 20 weeks. We report that rats eating the EPA-enriched diet exhibited a reduction of interstitial cardiac fibrosis and ameliorated LV diastolic dysfunction despite the continuous increase in blood pressure. However, we found that EPA did not have an impact on cardiac hypertrophy. Interestingly, the EPA diet increased mRNA expression of M2 macrophage marker Mrc1 and interleukin-10 in cardiac tissue. These findings indicated that the antifibrotic effects of EPA are mediated in part by phenotypic polarization of macrophages toward anti-inflammatory M2 macrophages and increases of the anti-inflammatory cytokine, interleukin-10. In summary, EPA prevents the exacerbation of cardiac fibrosis and LV diastolic dysfunction during sustained pressure overload. EPA could represent a novel treatment strategy for hypertensive cardiomyopathy.

Hypertension (HTN) affects ∼33% of the population and is a major risk factor for the development of heart failure (HF) (1, 2). Left ventricular (LV) remodeling induced by sustained pressure overload initially leads to concentric hypertrophy as a compensatory response followed by interstitial and perivascular cardiac fibrosis (3, 4). Robust clinical evidence demonstrates a strong association between cardiac fibrosis and adverse outcomes in patients with HF (5, 6, 7, 8). Importantly, pressure overload triggers inflammatory and biochemical responses that promote cardiac fibroblast conversion to generate excess extracellular matrix deposition and increased cardiac stiffness, inducing diastolic dysfunction (3). Despite these dire consequences, the ability to modulate cardiac fibrosis remains empiric and is currently limited to aggressive antihypertensive therapy (9, 10). Due to the essential role in cardiac remodeling and its irreversible nature, early modulation of cardiac fibrosis represents a crucial target for treating hypertensive heart disease.

Recent epidemiological and clinical trial data evaluating dietary omega-3 fatty acids have demonstrated beneficial cardiovascular (CV) effects (11). Purified eicosapentaenoic acid (EPA), one of the main forms of omega-3 fatty acids, consistently reduces CV events, superior to the standard of care (12, 13, 14). Evidence suggests that these beneficial effects, in addition to lower triglyceride levels, may be related to the anti-inflammatory properties of EPA (15). Pretreatment with EPA prevented LV dysfunction and interstitial cardiac fibrosis induced by acute pressure overload in mice (16). Similarly, EPA pretreatment also prevented cardiac fibrosis and hypertrophy in the noninfarcted areas postmyocardial infarction surgically induced in mice by promoting macrophage polarization towards anti-inflammatory M2 macrophages (17).

In this study, we considered whether purified EPA consumption prevents cardiac fibrosis, LV dysfunction, and myocardial inflammation in animals with established HTN, a situation similar to the common clinical condition of essential HTN.

## Materials and Methods

### Animals

All animal procedures and protocols were approved by the Wake Forest University Animal Care and Use Committee and conformed to the principles of the National Institutes of Health and Public Health Service Policy on Humane Care and Use of Laboratory Animals. Twelve-week-old male spontaneously hypertensive rats (SHRs) were purchased from Charles River (Wilmington, MA), housed under standard conditions, and fed experimental diets and tap water ad libitum.

### Diets

Each diet contained 4% test oil by weight; the EPA-enriched diet had 1.9 g of EPA plus 38.1 g corn oil (as vehicle) per Kg of rat chow (0.19% by weight) and the control (CTL) diet contained 40 g of corn oil per kg of rat chow (Dyets Inc., Bethlehem, PA). EPA was supplied to Dyets Inc. to manufacture rat chow and was >99% pure EPA measured by gas chromatography.

### In vivo studies

**Fig. 1:**
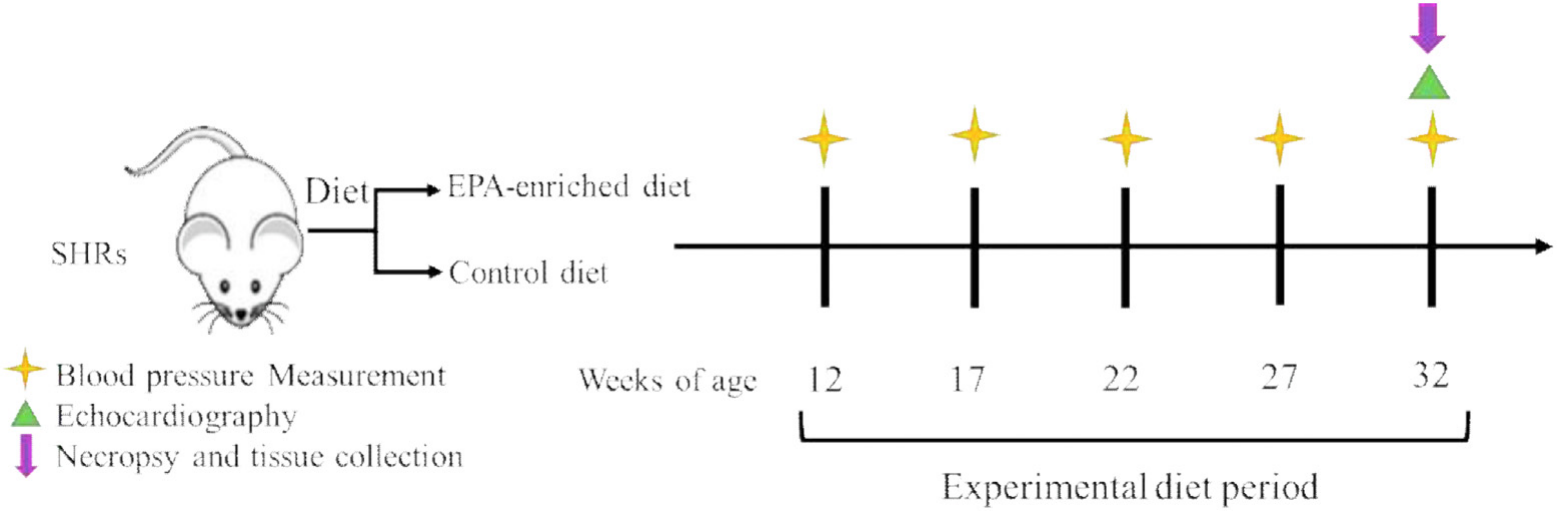
Study Design. Twelve-week-old SHRs were randomized to eat EPA-enriched or control diets for 20 weeks. Animals underwent blood pressure measurements every 5 weeks and prior to necropsy, echocardiographic evaluation of left ventricular (LV) structure and function was conducted. EPA, eicosapentaenoic acid; SHRs, spontaneously hypertensive rats.

Thirty 12-week-old SHRs were randomized to eat EPA-enriched (EPA rats) or CTL diet for 20 weeks (Fig. 1). The age of the rats and timepoints were chosen so that the diet was initiated upon the onset of HTN and the study concluded during a period of established HTN and associated myocardial remodeling (18). Systolic and diastolic blood pressures (SBP, DBP) were measured every 5 weeks during the experimental period by the noninvasive tail-cuff method. A minimum of three blood pressure (BP) measurements were recorded at each session and averaged. At the experimental endpoint, euthanasia was conducted following IP injection of sodium pentobarbital (50 mg/kg) by removal of the heart and lungs. The LV was separated from the right ventricle and weights were recorded. Lungs were weighed after removal of the pleura, trachea, and esophagus portions and blotted dry to obtain the wet weight and weighed again after drying in an oven at 60°C for 24 h. A short-axis, mid-section of the LV was fixed in 4% paraformaldehyde, and the apical portion was snap-frozen in liquid nitrogen and stored at -80°C for further biochemical analysis.

### Echocardiography

Assessments of the LV function were performed before necropsy, at the end of the experimental period (20 weeks) using a Vevo 2100 small animal echocardiographic system (VisualSonics Inc, Toronto, ON, Canada) by an experienced core echocardiographer who was blinded to the experimental groups. Rats were anesthetized with 1.5% isoflurane by continuous inhalation. Heart structure and function were assessed using two-dimensional, M-mode measurements of the LV posterior wall thickness, interventricular septum, and internal chamber diameter (LVID) at the end of diastole (*d*) and systole (*s*) from mid-papillary short-axis images. The LVIDs served to calculate the ejection fraction and fractional shortening as FS (%) = [(LVID*d*-LVID*s*)/LVID*d*] x 100 and EF (%) = [(LVID*d*^3^– LVID*s*^3^)/LVID*d*^3^] x 100. LV mass was calculated assuming a spherical LV geometry as LV mass= 1.04 ([LVID + LVPW + IVS]^3^- [LVID]^3^) -13,6 g. LV diastolic function was assessed using a standard two-dimensional B-mode ultrasound of the apical four-chamber view. Early mitral annular velocity (e’) and the ratio of early transmitral filling velocity-to-early mitral annular velocity (E/e’) were obtained using pulsed tissue Doppler imaging. The E/e’ ratios are used as a marker of LV filling pressure.

### Measurement of fatty acids in erythrocytes and cardiac tissue

Blood was collected from inferior vena cava after euthanasia and before removal of the heart for measurement of fatty acids in erythrocytes and in a subset of cardiac tissue (n = 6). Briefly, washed RBC and tissue samples (50 mg) underwent saponification with 0.1 ml 50% potassium hydroxide and 1 ml 95% ethanol (Warner-Graham Company) and heated at 60°C for 1 h. Optima hexanes (Fisher) were added to separate and remove the unsaponifiables. Fatty acids in the aqueous phase were acidified with 0.1 ml of glacial acetic acid and re-extracted back into hexane. Samples were then methylated with 0.5 N NaOH in methanol and BF_3_ at 100°C for 10 min, and fatty acids were extracted with isooctane. Isooctane was collected and analyzed by CP-Select for FAME (Varian) capillary gas chromatography column with a 0.25 um internal diameter (Agilent 6890N) to determine fatty acid distribution. Ionized fatty acid fragments were quantified by the total ion current. Sample levels of fatty acids were identified by comparison with a fatty acid methyl ester standard mixture (Supelco, Nu-Check Prep, and Matreya). ChromPerfect™ Spirit Chromatography Data System (version 5.0) was used to analyze the chromatographs.

### Plasma lipids

Concentration of total cholesterol, free cholesterol, and triglycerides were measured enzymatically using commercially available kits (Wako) as previously published (19).

### Collagen volume fraction and perivascular fibrosis

LV tissues were prepared in optimum cutting temperature compound-embedded frozen blocks, 5 μm thick, mid-ventricular sections were obtained and stained with picrosirius red (0.1% Sirius Red F3BA in picric acid). To assess interstitial cardiac fibrosis, 20 random microscopic fields were obtained, excluding vessels, using a 20X objective, and collagen volume fraction was expressed as a percent of the area as previously described (20, 21). Perivascular fibrosis was obtained from 8–20 short-axis images of intramural coronary arteries, and the perivascular collagen area was normalized to vessel luminal area (22). Interstitial and perivascular fibrosis was analyzed using Image J software (National Institute of Health, Bethesda, MA).

### Immunolabeling

Frozen LV sections (5 μm thick) were incubated in a pressure cooker with citrate buffer (Sigma-Aldrich, St Louis, MO) for rehydration and antigen retrieval. The tissue was labeled with CD68 antibody (1:50, BioRad, Hercules, CA) to identify macrophages and DAPI (Invitrogen, Waltham, MA) to identify nuclei. All sections were cover-slipped using ProLong™ Gold Antifade (Invitrogen). Six to ten images were acquired using confocal microscopy and the number of macrophages was quantified using Image J.

### Cardiac mast cell density

LV sections (5 μm thick) were stained with toluidine blue to quantify the total number of mast cells present in each cross-sectional LV as previously described (20) by a blinded observer (G. C. M.). Mast cell density was calculated by dividing the number of cells by the area of the section.

### Multiplex ELISA cytokine measurements

We employed a commercially available quantitative rat ELISA-based chemiluminescent inflammation assay kit (Quansys Biosciences Inc., West Logan, UT) that allowed simultaneous measurements of interleukin (IL)-1α, IL-1β, IL-2, IL-4, IL-6, IL-10, interferon-γ, and TNF-α using 25 μl of sample per well. Transforming growth factor (TGF)-β was measured with a commercially available rat ELISA kit (Abcam, Cambridge, United Kingdom). All cytokines were measured in LV tissue lysates and samples were run in duplicate and averaged.

### qPCR

Total RNA in tissues was extracted using Trizol (Invitrogen). Complementary DNA (cDNA) preparation and quantitative PCR (qPCR) were conducted as described previously. Primers are listed in Table 1.

**Table 1:** Forward and reverse primers used in qPCR

Gene Name	Forward Primer (5'→3′)	Reverse Primer (5'→3′)
*Arg1*	CCAGTATTCACCCCGGCTAC	GTCCTGAAAGTAGCCCTGTCT
*Cd68*	TGTTCAGCTCCAAGCCCAAA	GCTCTGATGTCGGTCCTGTTT
*Gapdh*	CCAAGGTCATCCATGACAACTT	AGGGGCCATCCACAGTCTT
*IL-1β*	CAGCTTTCGACAGTGAGGAGA	TTGTCGAGATGCTGCTGTGA
*IL-10*	CCTGGTAGAAGTGATGCCCC	AGACACCTTTGTCTTGGAGCTTAT
*MRC1*	GAGGACTGCGTGGTGATGAA	CATGCCGTTTCCAGCCTTTC
*TNF-α*	ATGGGCTCCCTCTCATCAGT	GCTTGGTGGTTTGCTACGAC

### Statistical analysis

All results are reported as mean ± SD. Results were analyzed using two-sample *t-*test, one-way ANOVA, as indicated. A *P*-value of <0.05 was considered significant. All analyses were completed using GraphPad Software. Pearson Correlation tests were also performed using the same software.

## Results

### Biometric parameters, BPs, and plasma lipids

**Fig. 2:**
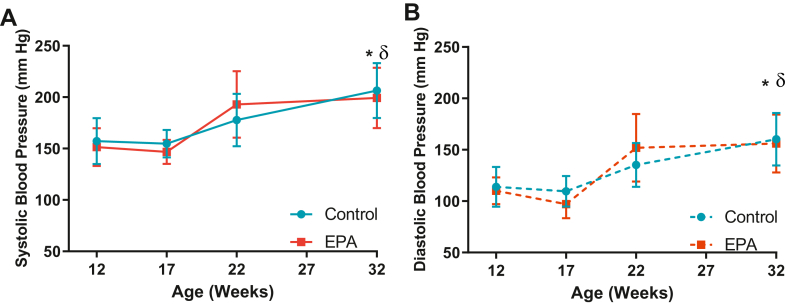
EPA did not influence the increases in blood pressure. Using the tail-cuff method, BP was monitored at baseline and then every 5 weeks. (A) systolic BP (solid lines) and (B) diastolic BP (dotted lines); SHRs on EPA-enriched (red) or control (blue) diets. Graphs show mean ± SD. ∗*P* < 0.001 versus baseline BP (12 weeks) of SHRs on control diet and δ *P* < 0.001 versus baseline BP of animals on EPA-enriched diet. BP, blood pressure; EPA, eicosapentaenoic acid; SHRs, spontaneously hypertensive rats.

Twelve-week-old SHRs exhibit early stages of pathologic cardiac remodeling including hypertrophy and fibrosis induced by a sustained increase of BP (18). To determine whether the protective effects of EPA in our cohort of hypertensive animals were influenced by changes in BP or any other biometric parameters, body weight and blood pressure were monitored and postnecropsy key organ weights were determined. Results for biometric parameters are presented in Table 2. There were no statistically significant differences in body weight, LV, right ventricle, or lung weights between groups (*P* > 0.05). At 12 weeks of age (baseline), there were no differences in BP between the groups (SBP, CTL: 157.3 ± 5.8 mmHg vs. EPA: 151.5 ± 4.7 mmHg, *P* = 0 .45, DBP, CTL: 113.92 ± 4.99 mmHg vs. EPA 110.1 ± 3.3 mmHg, *P* = 0.527, Fig. 2A, B). The SHRs from both the CTL and EPA groups increased their SBPs and DBPs throughout the experimental period and levels reached statistical significance at 32 weeks compared to baseline (*P* < 0.0001, Fig. 2A, B). No differences were found between the groups at any time point. Plasma lipids are presented in Table 3. There were no statistical differences for total plasma cholesterol, cholesterol esters, or triglycerides between groups. While free cholesterol tended to be increased in rats eating EPA-enriched chow, it did not reach statistical significance (*P* = 0.08).

**Table 2:** Biometric parameters

Groups	Control (n = 14)	EPA (n = 15)	*P* Value
BW, g	405 ± 19	401 ± 36	0.35
LV, mg	1171.93 ± 69.8	1137.0 ± 92.5	0.13
LV/BW, mg/g BW	2.9 ± 0.1	2.8 ± 0.2	0.19
RV, mg	213.2 ± 26.5	224.1 ± 25.7	0.14
RV/BW, mg/g BW	0.5 ± 0.1	0.6 ± 0.1	0.15
Lung _w_, mg	1422.1 ± 61.8	1422.7 ± 87.2	0.49
Lung _w_/BW, mg/g BW	3.52 ± 0.16	3.57 ± 0.31	0.28
Lung _d_, mg	300.3 ± 19.8	300.7 ± 31.7	0.49
Lung _w/d_, mg	0.21 ± 0.02	0.21 ± 0.01	0.40

All of the values are mean ± SD. BW indicates body weight; LV, left ventricle; RV, right ventricle; Lung _w_, wet lung; Lung _d_, dry lung.

**Table 3:** Plasma lipids

Groups	Control (n = 6)	EPA (n = 6)	*P* Value
Total plasma cholesterol (mg/dl)	53.2 ± 11.7	61.8 ± 10.3	0.20
Free cholesterol (mg/dl)	10.2 ± 3.2	13.5 ± 2.8	0.08
Cholesteryl esters (mg/dl)	43.1 ± 8.9	48.3 ± 8.2	0.30
Triglycerides (mg/dl)	45.5 ± 14.5	43.0 ± 26.6	0.84

Values reported as mean ± SD.

### EPA-enriched diet increased EPA levels in RBC membrane but not in cardiac tissue

**Fig. 3:**
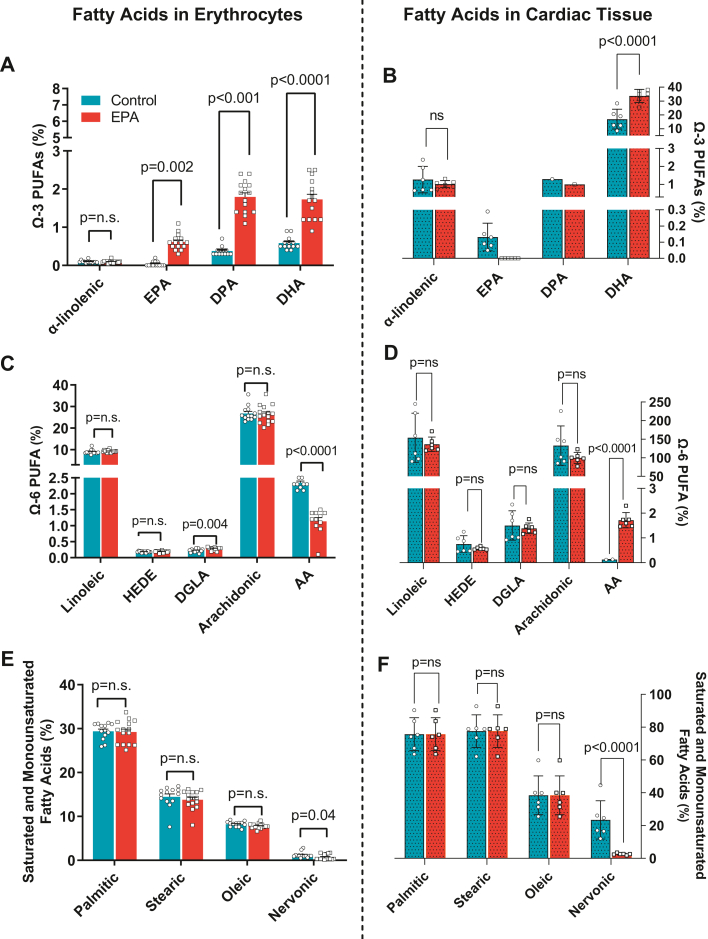
EPA-enriched diet increased levels of EPA in erythrocytes but not in cardiac tissue. Fatty acid content in erythrocytes (left panels A, C, E) and cardiac tissue (right panel B, D, F) of rat on control (blue bars) or EPA-enriched diets (red bars). (A, B) omega(Ω)-3 PUFAs, (C, D) omega(Ω)-6 PUFAs, and (E, F) saturated and monounsaturated fatty acids,. n.s. indicates nonsignificant difference. AA, adrenic acid; DPA, docosapentaenoic acid; DHA, docosahexaenoic acid; DGLA, diacylglycerol lipase alpha; EPA, eicosapentaenoic acid; HEDE, eicosadienoic acid; PUFA, polyunsaturated fatty acid.

We measured fatty acid levels (percent mass of total FAs) in erythrocytes and cardiac tissue in response to dietary EPA. Previous reports demonstrate that supplementation of EPA for 12 weeks in mice induced an increase in EPA in erythrocytes similar to patients treated with omega-3 PUFAs and that EPA does not increase in cardiac cells (16, 23). As expected, the rats fed the EPA diet exhibited higher erythrocyte EPA fatty acid levels than rats on the CTL diet (CTL: 0.007 ± 0.02% vs. EPA: 0.61 ± 0.20%, *P* < 0.001, Fig. 3A). Similarly, EPA-fed animals had higher levels of docosapentaenoic acid (DPA) (CTL: 0.38 ± 0.12% vs. EPA: 1.80 ± 0.41%, *P* < 0.001, Fig. 3A) and docosahexaenoic acid (DHA) (CTL: 0.60 ± 0.16% vs. EPA: 1.73 ± 0.52% *P* < 0.001, Fig. 3A). Interestingly, EPA was not detectable in cardiac tissue of EPA-fed animals (CTL: 0.13 ± 0.08% vs. EPA: not detectable, Fig. 3B), and DPA was only detected in one sample from each group and, similarly to erythrocytes, DHA was significantly higher (CTL: 16.9 ± 7.1% vs. EPA: 33.7 ± 4.7%, *P* < 0.0001, Fig. 3B). Amongst omega-6 fatty acids, DGLA was significantly increased in EPA in erythrocytes of EPA-fed animals (CTL: 0.22 ± 0.05% vs. EPA: 0.28 ± 0.04%, *P* = 0.004, Fig. 3C) but did not change in cardiac tissue (CTL: 1.50 ± 0.59% vs. EPA: 1.39 ± 0.22%, p = ns, Fig. 3D). Arachidonic acid was significantly decreased in both erythrocytes and cardiac tissue of EPA-fed rats (*P* < 0.001)(Fig. 3C, D). There were no changes in saturated and monounsaturated fatty acids except for nervonic acid which was decreased in both RBCs and cardiac tissue from EPA-fed rats (RBC, CTL: 1.20 ± 0.66% vs. EPA: 0.69 ± 0.63% *P* = 0.04; cardiac tissue, CTL: 23.4 ± 11.64% vs. EPA: 2.61 ± 0.53%, *P* < 0.0001, Fig. 3E, F).

### EPA ameliorates HTN-induced LV interstitial myocardial fibrosis and preserves LV diastolic function

**Fig. 4:**
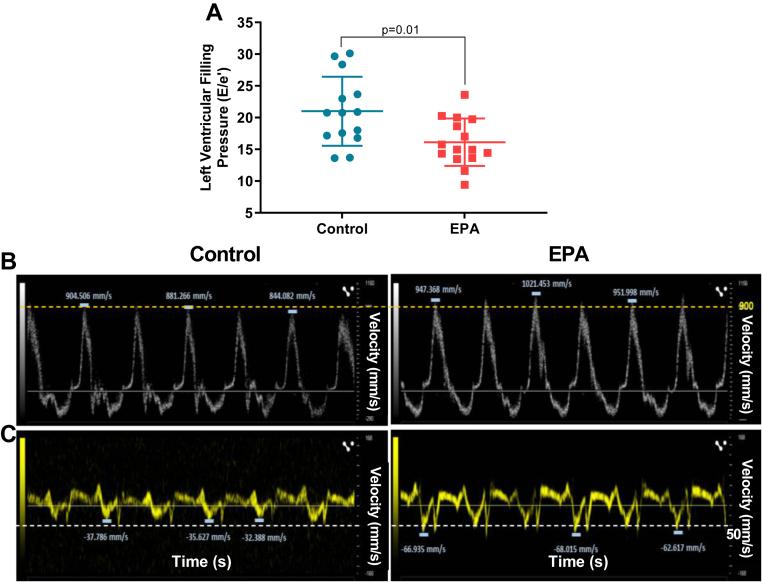
Left ventricular function was preserved in SHRs on EPA diet. (A) left ventricular filling pressures (E/e’) of rats on control- (blue dots) or EPA-enriched (red squares) diets. Representative Doppler images of the (B) transmitral flow and (C) mitral annulus tissue velocities. All values are mean ± SD. EPA, eicosapentaenoic acid; SHRs, spontaneously hypertensive rats.

**Fig. 5:**
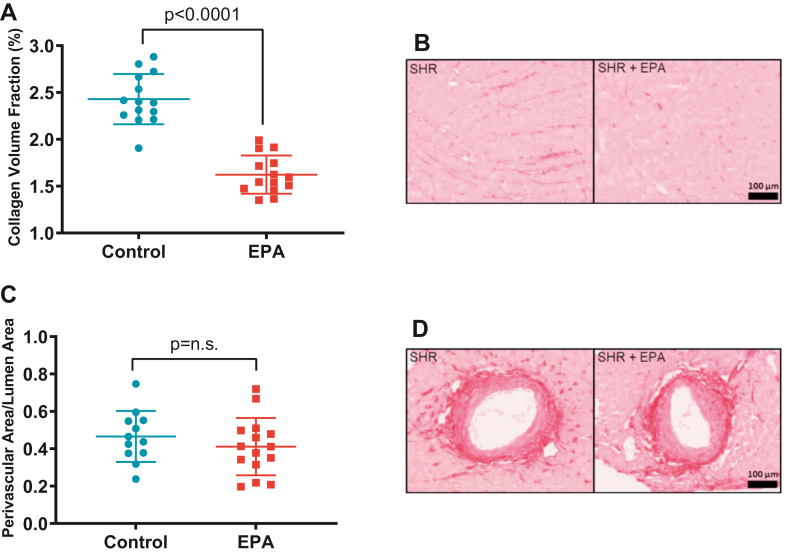
EPA-ameliorated interstitial cardiac fibrosis. Scattered dot plots showing (A) left ventricular interstitial collagen volume fraction and (C) perivascular collagen. n.s. indicates nonsignificant difference. Representative microphotographs of picrosirius red-stained left ventricle depicting (B) interstitial and (D) perivascular collagen. EPA, eicosapentaenoic acid;

**Fig. 6:**
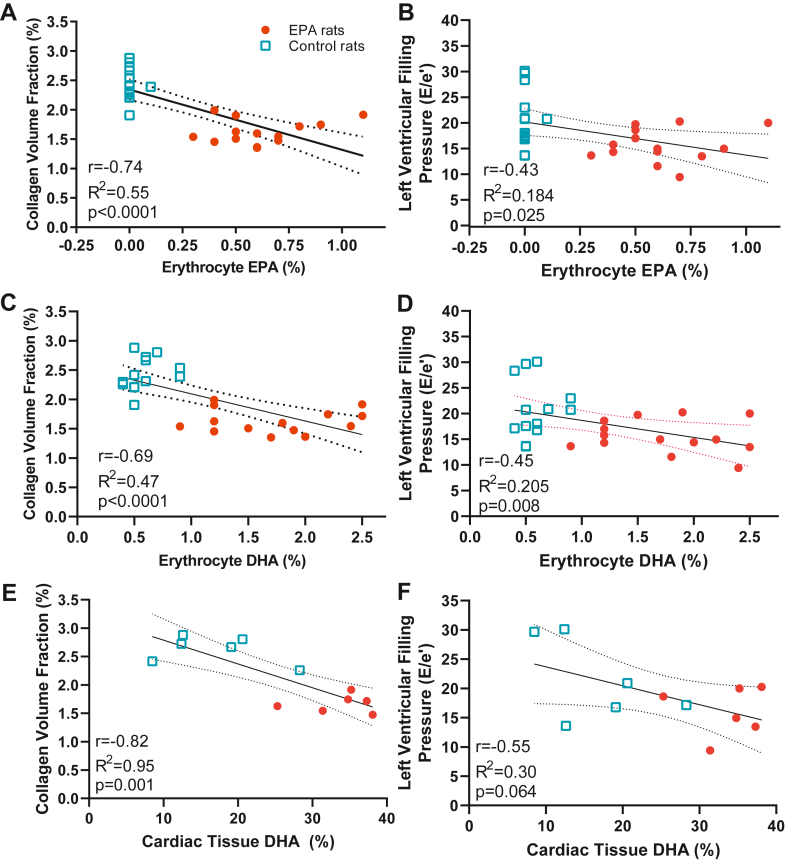
EPA and DHA levels were inversely associated with cardiac fibrosis and left ventricular filling pressures. Association between collagen volume fraction (CVF) and (A) erythrocyte EPA (%), (C) erythrocyte DHA (%), (E) cardiac tissue DHA (%) and association between left ventricular filling pressure and (B) erythrocyte EPA (%), (D) erythrocyte DHA (%), (F) cardiac tissue DHA (%). DHA, docosahexaenoic acid; EPA, eicosapentaenoic acid.

LV pressure overload results in cardiac interstitial and perivascular fibrosis and hypertrophy (24) which contributes to increased cardiac stiffness. Progression of these two pathologic conditions will evolve into HF (25, 26). While EPA had no effects in LV structure or systolic function (*P* > 0.05, Table 4), animals fed the EPA supplemented diet exhibited significantly lower LV diastolic filling pressures than the rats on the control diet (E/e’, CTL: 21.0 ± 1.24 vs. EPA: 16.2 ± 1.20 , *P* = 0.01; Fig. 4A) and exhibited less LV interstitial collagen deposition than those eating the control diet (CTL: 2.43 ± 0.26% vs. EPA: 1.62 ± 0.20%, *P* < 0.0001; Fig. 5A, B). We found no differences in perivascular collagen deposition/lumen area ratio between groups (CTL: 0.48 ± 0.31 vs. EPA 0.46 ± 0.3, *P* = 0.17, Fig. 5C, D). Furthermore, we explored the relationship between LV diastolic filling pressure and collagen volume fraction with erythrocyte and cardiac tissue amount of EPA and DHA levels. We found that both erythrocyte and cardiac levels of EPA and DHA were inversely correlated with diastolic function and cardiac fibrosis (Fig. 6A–F).

**Table 4:** Echocardiographic parameters

Groups	Control (n = 14)	EPA (n = 15)	*P* Value
Heart rate, bpm	350 ± 23	355 ± 39	0.33
LVPW_d_, mm	2.52 ± 0.34	2.47 ± 0.51	0.39
LVID_d_, mm	5.97 ± 0.68	5.63 ± 0.51	0.07
IVS_d_, mm	1.99 ± 0.25	2.10 ± 0.30	0.15
LV mass, mg	796 ± 159	746 ± 138	0.18
EF, %	74.8 ± 8.8	72.5 ± 9.8	0.25
FS, %	45.2 ± 8.6	43.0 ± 8.9	0.25

Values reported as mean ± SD. LVPW_d_, left ventricle posterior wall in diastole; LVID_d_, left ventricular internal diameter in diastole; IVS_d_, interventricular septum in diastole; EF, ejection fraction; FS, fractional shortening.

### EPA changed LV macrophage gene signature in response to HTN with no effect on total number of macrophages

**Fig. 7:**
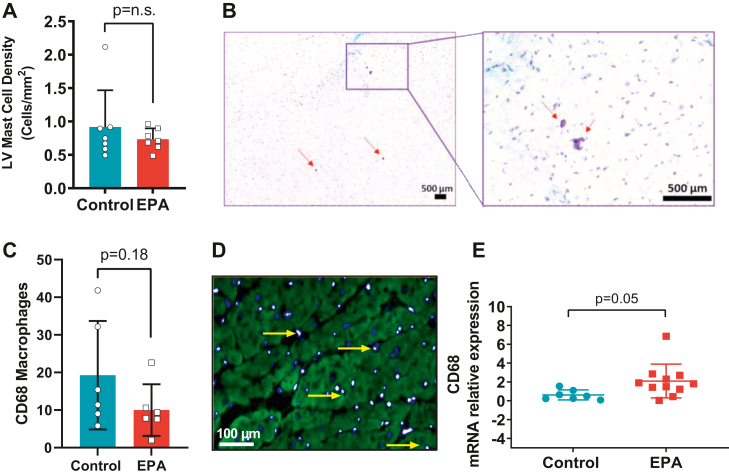
EPA Changed LV Macrophage Gene Signature in Response to HTN. Quantification of (A) mast cell density and (C) macrophages per LV section and (E) CD68 mRNA in left ventricular tissue of rats on EPA-enriched (red bars/squares) or control diet (blue bars/circles). Representative images for (B) mast cells (red arrows) (D) macrophages (CD68^+^, yellow arrow shows costaining with DAPI). Data are expressed as mean ± SD. n.s. indicates nonsignificant difference. EPA, eicosapentaenoic acid; HTN, hypertension; LV, left ventricular.

**Fig. 8:**
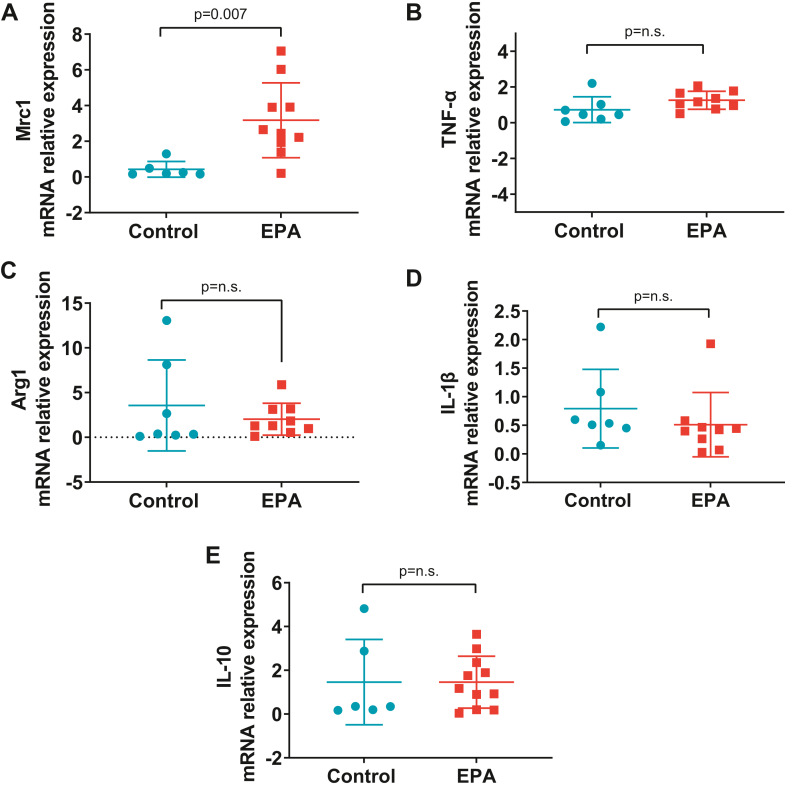
EPA induced increase in M2 macrophage mannose receptor *Mrc1*. mRNA expression of LV markers of (A) Mrc1, (B) TNF-α, (C) Arg1, (D) IL-1β, and (E) IL-10 analyzed by real-time polymerase chain reaction post diet. Data are presented as mean ± SD. n.s. indicates nonsignificant difference. EPA, eicosapentaenoic acid; IL, interleukin; LV, left ventricular; TNF, tumor necrosis factor.

Pressure overload promotes the recruitment of mast cells (27) and macrophages in cardiac tissue, favoring polarization towards proinflammatory M1 macrophages (28). EPA administration had no effect on mast cell density (CTL: 0.92 ± 0.20 vs. EPA: 0.73 ± 0.06, *P* = 0.20, Fig. 7A, B). Our data shows no significant differences in the total number of macrophages identified by labeling with CD68 (CTL: 19.27 ± 14.41 vs. EPA: 10.0 ± 6.8, *P* = 0.18, Fig. 7C, D). However, we found CD68 mRNA expression to be significantly increased in cardiac tissue of EPA-fed rats (CTL: 0.6 ± 0.5 vs. EPA: 2.1 ± 1.8, *P* = 0.05, Fig. 7E). Furthermore, mRNA *Mrc1* expression was significantly increased in the EPA group, denoting a shift towards M2 macrophage phenotype (CTL: 0.4 ± 0.4 vs. EPA: 3.1 ± 2.1 v, *P* = 0.007, Fig. 8A). There were no differences between groups in the mRNA expression of *TNF-α*, *Arg1*, *I*L*-1β*, or *I*L*-10* (Fig. 8B–E, respectively).

### IL-10 increased in the myocardial tissue of EPA rats

**Fig. 9:**
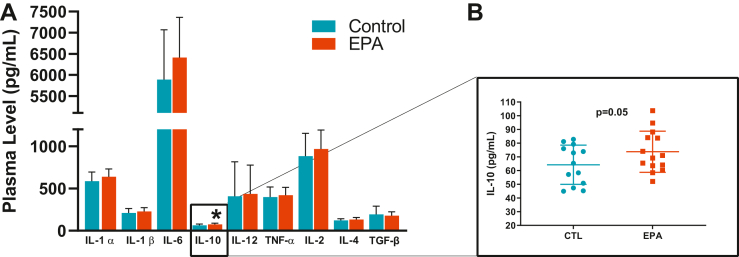
EPA induced an increase in anti-inflammatory cytokine IL-10 in cardiac tissue. Myocardial levels of (A) cytokines in rats on the EPA-enriched (red bars) or control diet (blue bars). EPA induced a significant increase of interleukin-10 (B). All of the values expressed as mean ± SD. EPA, eicosapentaenoic acidIL, interleukin.

In order to determine whether inflammatory responses are involved in the improved cardiac structure and function of EPA rats, levels of multiple proinflammatory and anti-inflammatory cytokines were measured in the LV tissue. Anti-inflammatory cytokine IL-10 was found to be significantly increased in LV homogenates of EPA rats (CTL: 64.23 ± 14.29 pg/ml vs. EPA: 73.7 ± 15.0 pg/ml, *P* = 0.05, Fig. 9). All other cytokines did not exhibit group differences. Furthermore, levels of TGF-β, a known profibrotic cytokine, were not significantly different between the two diet groups.

## Discussion

The current study contributes to the growing evidence that consumption of EPA (C20:5 n-3) may be an effective strategy to mitigate cardiac fibrosis and LV dysfunction. Employing a clinically relevant model of established HTN, we demonstrated that EPA consumption 1) mitigated the progression of HTN-induced interstitial cardiac fibrosis and associated LV diastolic dysfunction, 2) promotes the polarization of macrophages toward anti-inflammatory M2 macrophages, and 3) increased cardiac levels of the anti-inflammatory cytokine IL-10.

In 2019, icosapent ethyl, a highly purified formulation of EPA was approved by the U.S. Food and Drug Administration as adjuvant therapy for patients with hypertriglyceridemia (> 150 mg/dl), after the results of the Reduction of Cardiovascular Events with Icosapent Ethyl-Intervention Trial demonstrated a 25% reduction in cardiovascular disease events in high-risk patients (12). While the underlying mechanisms of these clinical benefits are not fully understood, there is an increasing interest in the pleiotropic anti-inflammatory effects of EPA that may enhance CV protection. Using a mouse model, two studies previously revealed that pretreatment with EPA attenuated cardiac fibrosis and remodeling induced by myocardial infarction and pressure overload (16, 17), supporting EPA supplement as potentially preventive therapy of cardiac remodeling. Our study further explores the efficacy of EPA as a treatment strategy to prevent the progression of the fibrotic response and cardiac fibroblast activation in the setting of persistent high-pressure overload, a frequently encountered clinical scenario. SHRs started EPA diet at 12 weeks old, an age at which this rat strain exhibits elevated SBP (>150 mmHg) compared to age- and gender-matched rats (18) and early stages of LV cardiac fibrosis (29, 30). EPA did not prevent the sustained increase of SBP and DBP during the experimental period (Fig. 2). As expected, rats consuming an EPA-enriched diet had a higher percentage of EPA in erythrocytes, despite a relatively lower levels than those previously reported in mice (16); additionally, erythrocyte EPA was elongated to DPA (C22:5 n-3) and DHA (C22:6 n-3) in the diet-fed rats. Interestingly, EPA was not detectable in cardiac tissue of rats fed with the EPA-enriched diet (Fig. 3B) but DHA on the other hand was significantly increased which suggests that EPA can rapidly and very efficiently be converted to DHA.

Our study demonstrated that EPA diet supplementation over a relatively short 20-weeks experimental period resulted in 33% less HTN-induced interstitial cardiac fibrosis despite the persistent increases in BP. This decrease resulted in a significant improvement of LV filling pressures (Fig. 4), and both levels of EPA and DHA in erythrocytes were inversely associated with LV filling pressures and concomitant myocardial fibrosis (Fig. 6A–D). However, because EPA levels were undetectable in cardiac tissue of EPA fed rats (Fig. 3B) and in turn, DHA was significantly increased, it is highly likely that DHA may be the main contributor to the preservation of the filling pressures and fibrosis in our model. Our findings are consistent with the reports by Eclov *et al.* that demonstrated that cardiac fibrosis induced by *trans*-aortic constriction was ameliorated in animals treated with EPA but not those treated with DHA (16). Similar to our results, they demonstrated that dietary supplementation with EPA failed to increase EPA levels in cardiac cells and that DHA integrates into the membranes of cardiac fibroblasts and cardiomyocytes.

Additionally, we did not observe changes in LV systolic function in either group. This is likely due to the fact that SHRs develop compensated LV concentric hypertrophy with preserved systolic function during the first year of age and progressively develop heart failure with reduced ejection fraction by 15 months of age (31, 32), a time point beyond our experimental observation. EPA did not have an impact on perivascular fibrosis; this is likely because this type of fibrosis is highly associated with elevated vascular pressure, which in our animals, it was constantly elevated. While clinical and preclinical studies have demonstrated that n-3 fatty acids consistently reduce triglyceride levels, they have also shown to increase LDL cholesterol (33, 34); the use of EPA alone (4 g/day) does not impact plasma cholesterol. Nonetheless, in our animal trial, plasma lipids were investigated and we found that EPA had no effect on LDL cholesterol (Table 3), confirming that EPA remains a safe prescription.

While activated cardiac fibroblasts are the culprit cells implicated in collagen deposition resulting in cardiac fibrosis, inflammatory cells play an important role in the regulation of the fibrotic response. Prior evidence suggests that EPA diet decreases polarization towards proinflammatory M1 macrophages in the infarcted myocardium and ameliorated cardiac postmyocardial infarction cardiac fibrosis and remodeling (17). In pressure overload conditions, expansion of cardiac macrophages causally contributes to the development of diastolic dysfunction and fibrotic remodeling in mice and humans (28); in our study, EPA had no effect on the total number of macrophages. However, CD68 mRNA levels exhibited a trend to be increased and interestingly, *Mrc1*, a marker of M2 macrophages (35, 36), was increased in cardiac tissue suggesting a shift toward an anti-inflammatory phenotype. Traditionally, M1-activated macrophages express proinflammatory cytokines, while M2 macrophages contain high levels of IL-10 (5), a cytokine known to inhibit the synthesis of proinflammatory cytokines via STAT3 activation (37). We found that EPA promoted higher levels of IL-10 in cardiac tissue, but proinflammatory and profibrotic cytokines such as IL-6 and TGF-β (21) were unchanged. These results are consistent with the report by Satoh-Asahara *et al.* that demonstrate that IL-10 expression in circulating monocytes increases in obese patients with dyslipidemia after 3 months of treatment with EPA (1.8 g daily); interestingly, these patients exhibited a decrease in arterial stiffness (38). IL-10 is well known for its anti-inflammatory actions (39), however, its role in fibrogenesis remains controversial. In an in vivo study where pathologic cardiac remodeling was induced by nephrectomy, aldosterone infusion, and high salt consumption, IL-10 produced by macrophages activated cardiac fibroblasts and increased collagen deposition and diastolic dysfunction (28). In contrast, a recent study by Verma *et al.* suggests that IL-10 inhibits phenoconversion of cardiac fibroblasts into myofibroblasts in pressure overload conditions (40). We speculate that in our study, EPA-induced increases of IL-10 may have offset the activation of profibrotic pathways by proinflammatory cytokines. Finally, a few studies have reported that specialized lipid mediators such as resolvins, protectins, and maresins (41) that originate from EPA and DHA may have antifibrotic effects (42, 43) and promote M2 phenotype polarization (44). We investigated whether anti-inflammatory resolving D1, resolving E1, and lipoxin A4-derived from EPA, DHA, and arachidonic acid, respectively, may be upregulated in LV tissue of EPA-fed rats. While all three standards for proresolvin mediators were detected at a concentration of 50 ng/ml, they were not detected in the samples (data not shown). More studies that are comprehensive and aimed at identifying specialized lipid mediators derived from EPA and DHA with therapeutic against cardiac fibrosis are needed to better understand the antifibrotic mechanisms of EPA in HTN.

Our observations may have important translational implications in the management of HTN. Recently, the results of the Systolic BP Intervention Trial (SPRINT) (9) revealed that intense BP control does not reduce cardiac remodeling or fibrosis and underscores the need for novel adjuvant therapies beyond BP control to mitigate HTN-induced fibrotic remodeling and prevent the progression of HF. The robust effects of EPA in attenuating cardiac fibrosis represent a novel approach in the treatment of hypertensive cardiomyopathy.

Our study should be interpreted in the context of the following limitations: first, we did not conduct long-term experiments to determine if mitigation of cardiac fibrosis by EPA will delay or prevent the transition into heart failure with reduced ejection fraction that occurs at ∼15 months of age in SHRs; second, we did not conduct studies in normotensive animals, consequently, we cannot definitively assess whether EPA ameliorates or protects from myocardial fibrosis and diastolic dysfunction; and third, we evaluated the effects of EPA independent from HTN medications. Further studies focusing on the antifibrotic effects of cardioprotective medications and EPA are warranted.

In conclusion, using a clinically relevant animal model of chronic HTN, we demonstrated that EPA supplementation mitigates the progression of cardiac fibrosis and associated LV diastolic dysfunction, potentially through modulating proinflammatory M1 macrophages. Further studies are needed to establish definitively the causal relationship between EPA administration and the anti-inflammatory phenotypic changes in macrophages and amelioration of cardiac fibrosis. Overall, our study adds to the growing evidence that purified EPA may be a promising therapeutic strategy for hypertensive cardiomyopathy.

## Data Availability

All data has been presented in this manuscript.

## Conflict of Interest

Amarin provided minor editorial suggestions to the final manuscript however, they did not participate in the study design, the execution of the experiments, data analysis and interpretation, or the writing of the manuscript.
